# Local Ecological Knowledge on Climate Change and Ecosystem-Based Adaptation Strategies Promote Resilience in the Middle Zambezi Biosphere Reserve, Zimbabwe

**DOI:** 10.1155/2019/3069254

**Published:** 2019-03-11

**Authors:** Olga Laiza Kupika, Edson Gandiwa, Godwell Nhamo, Shakkie Kativu

**Affiliations:** ^1^Chinhoyi University of Technology, School of Wildlife Ecology and Conservation, Private Bag 7724, Chinhoyi, Zimbabwe; ^2^Exxaro Chair in Business & Climate Change, Institute for Corporate Citizenship, University of South Africa, P.O. Box 392, UNISA 0003, Pretoria, South Africa; ^3^Department of Biological Sciences, Faculty of Science, University of Zimbabwe, Harare, Zimbabwe

## Abstract

Understanding local community perceptions on impacts, causes, and responses to climate change is vital for promotion of community resilience towards climate change. This study explored local ecological knowledge (LEK) held by local communities on climate change trends and impacts in the Middle Zambezi Biosphere Reserve (MZBR), Zimbabwe. The objectives of the study were to (i) investigate local community perceptions on trends and causes of climate change, (ii) identify biophysical impacts of climate change at the local level, and (iii) explore the ecosystem-based adaptation strategies towards climate change. The study used a mixed methods approach where a household questionnaire survey (*n*=320), key informant interviews (*n*=12), and focus group discussions (*n*=8) were used to collect data between April 2015 and October 2016. Results from the study show that local communities have observed decreasing rainfall and increasing temperatures as key indicators of climate change. Local communities observed water scarcity, changes in vegetation phenology, livestock and wildlife mortalities, and food shortages due to drought as the major impacts on their livelihoods. LEK can contribute to adaptive management strategies that enhance resilience of socioecological systems (SES) in the face of climate change by providing information on the status and use of biophysical components of the environment and by highlighting potential local adaptation strategies that can sustain key livelihood practices.

## 1. Introduction

Communities from different parts of the world use local knowledge about ecosystems to recognize and respond to the impacts of climate change and variability [1]. African rural communities have been documented as constructing climate change realities based on their experiences of the impacts and effects [2]. Although the observation of global climate change has been largely based on meteorological data, there is paucity of information on how humans use local ecological knowledge to recognize and respond to such changes [3]. This calls for research to explore the role of local culture in identifying and responding to threats imposed by the changing climate. Adger et al. [4] suggest that local communities could interpret and construct climate change trends and local indicators within a cultural setting. The United Nations (UN) recognizes the significant role played by indigenous knowledge, cultures, and traditional practices in promoting sustainable development, equity, and management of the environment [5]. Adger et al. [4] further argue that since culture is embedded in societal modes of production, consumption, lifestyles, and social organization, it should be recognized in understanding both mitigation and adaptation to climate change. Understanding and building upon perceptions, experiences, and IK on climate change can contribute towards strengthening the resilience of poor societies who are characterized by weak infrastructure and economic well-being [6].

Traditional knowledge is derived from indigenous knowledge systems (IKSs), the designation that is used to refer to the modus operandi and processes that the indigenous peoples use to harness local knowledge [7]. Local/traditional/indigenous traditional knowledge is defined as the intellectual behavior and beliefs of indigenous societies or local information about the relationship of living beings (including humans) with one another and with their environment [8]. The knowledge exists and is developed, accumulated, and transmitted culturally through local community experiences and know-how across generations [8, 9]. Also, known as indigenous technical science, indigenous knowledge is dynamic and is informed by local communities' interactions with their local biophysical and social environment [10]. Indigenous knowledge encompasses interrelated subsets of local ecological knowledge (LEK), seasonal knowledge, and phenological knowledge [11].

Local ecological knowledge (LEK) refers to knowledge, practices, and beliefs shared among local resource users regarding ecological interaction within ecosystems [12]. LEK comprises people's lived experiences and their dialectical interaction with the natural environment [13]. Thus, local knowledge is a key component of integrated ecosystem and community-based adaptation strategies. The Convention of Biological Diversity (CBD) [14] defines ecosystem-based adaptation (EbA) as the use of biodiversity and ecosystem services in an overall adaptation strategy. Reid et al. [15] defines community-based adaptation as a community-led process, based on communities' priorities, needs, knowledge, and capacities, which should empower people to plan for and cope with the impacts of climate change. EbA includes approaches such as the sustainable management, conservation, and restoration of ecosystems to provide services that help people adapt to the adverse effects of climate change [14]. Approaches to ecosystem-based adaptation take into account the multiple social, economic, and cultural cobenefits for local communities as part of an overall adaptation strategy [16]. This study investigated LEK inclusive of seasonal ecological knowledge (SEK) on climate trends and responses to climate change impacts in the context of a biosphere reserve.

The United Nations Educational, Scientific and Cultural Organization (UNESCO) Man and Biosphere Reserves (MAB) are established directly to bring together biodiversity, cultural diversity, and ecosystem services, thus promoting ecological security and models for sustainable development [17]. UNESCO MAB, together with its World Network of Biosphere Reserves (WNBR) functions as a Global Observatory for Climate Change Mitigation and Adaptation focused on promoting integrated monitoring, multidisciplinary approaches, and participatory activities, supporting climate change management [18]. The WNBR strives to implement the Paris Agreement and United Nations (UN) Sustainable Development Goals (SDGs) by fostering the improvement of human livelihoods and protection of natural and managed ecosystems for sustainable development [19]. Yashina [20] notes that biosphere reserves play an important role in developing and implementing mitigation and adaptation measures as stipulated in the Madrid Climate Change Strategy Action Plan Framework of 2008 and the Climate Change Initiative of 2009. Target 24 of the Action Plan predicts that biosphere reserves can be used as learning sites for research into, adaptation to, and mitigation of climate change impacts [20]. UNESCO [21] calls for member states to respond to new kinds of conservation challenge posed by climate change, developing innovative policy, tailoring management strategies, and recognizing the value of resilient protected area systems that help safeguard the global environment and human societies from the threats posed by climate change. UNESCO MAB strategy highlights the need for local communities to integrate indigenous knowledge in the fight against climate change. Thus, embracing indigenous knowledge in climate change adaptation is vital to enhance community resilience of communities to climate change [22] in biosphere reserve settings.

Several studies have focused on the impacts and small-scale farmers' adaptation towards climate change and variability at the agriculture-wildlife interface in Zimbabwe [23, 24]. Nyikahadzoi et al. [25] studied the factors influencing climate change adaptation among small-scale farmers in Hurungwe District, Zimbabwe. Ndebele-Murisa et al. [26] studied the effects of climate change and variability on fisheries-based livelihoods in Kariba, Zimbabwe. However, a few studies have focused on the role of local knowledge in understanding climate change trends, impacts, and adaptive strategies among agroecological-based livelihoods within the MZBR. This study uses two case studies to explore local ecological knowledge (LEK) held by local communities on climate change trends, impacts, and adaptation in the Middle Zambezi Biosphere Reserve (MZBR), Zimbabwe.

The growing body of knowledge on local indigenous knowledge about climate change impacts on biophysical systems provides novel contributions towards our understanding of local climate change and people's responses [27–29] in rural communities who are determined to develop sustainable environments [6]. Murphy et al. [30] contend that successful adaptation to climate change requires understanding processes of social and biophysical change and their interactions within socioecological systems. Thus, LEK can be used to understand community adaptive practices which promote resilience to environmental changes [31] that have negative effects on local livelihoods and sustainable development [10]. LEK can therefore supplement scientific data by providing primary and comprehensive descriptions of the biophysical and socioeconomic components of the biosphere landscapes that are experiencing climate change stresses [20]. In addition, local community traditional daily practices such as weather forecasting can provide a myriad of benefits including making informed decisions to enhance agricultural food security [11]. The objectives of this study were to (i) investigate local community perceptions on trends and causes of climate change, (ii) identify biophysical impacts of climate change at the local level, and (iii) explore the ecosystem-based adaptation strategies towards climate change on the MZBR, Zimbabwe.

## 2. Materials and Methods

### 2.1. Study Sites

**Figure 1:**
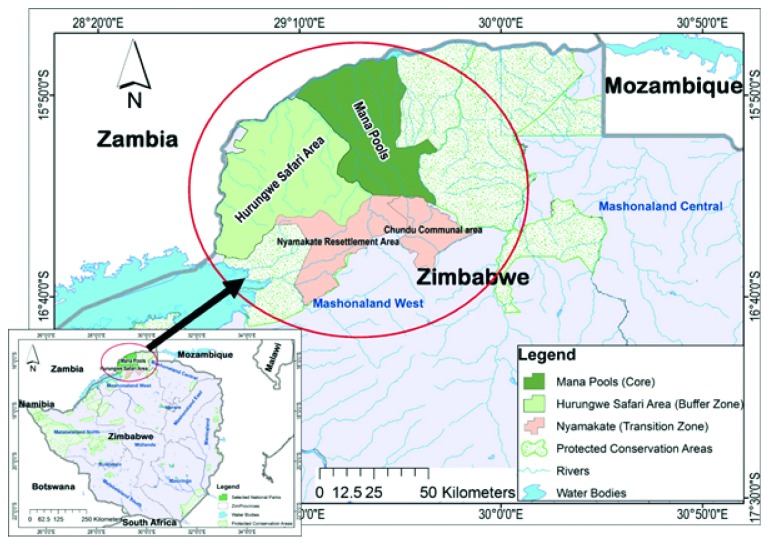
Location of Nyamakate Resettlement and Chundu Communal Area in the Middle Zambezi Biosphere Reserve (source: authors).

The study was conducted in the Chundu Communal and Nyamakate Resettlement Area (Table 1) located in the transitional zone of the Middle Zambezi Biosphere Reserve (MZBR), in the northern margins of Mashonaland West Province, Zimbabwe. The case study communities were purposively sampled due to their proximity to Mana Pools National Park and Hurungwe Safari Area (Figure 1) as an important factor in their recruitment. The study area is made up of indigenous people who were forced to migrate from the Zambezi Valley prior to the establishment of the adjacent protected areas, namely, Hurungwe Safari Area, Mana Pools National Park, and Charara Safari Area (Figure 1).

The Nyamakate chiefdom and Chundu Communal Area was established in the Zambezi valley before and after colonization by the white men, respectively [33]. However, the chiefdom was dismantled by the colonial government and the occupation of the contemporary Nyamakate Resettlement was done in the 1980s [32, 33]. Nyamakate Resettlement and Chundu Communal Areas lie within agroecological region 3. Chimhowu and Hulme [32] observed that most agricultural seasons experience drought in the area as indicated by the 1981–82, 1982–83, 1983–84, 1986–87, 1991–92, 1994–95, and 1996–97 droughts. The 1991–92 drought was the most severe, and it had profound effects on livelihoods [32].

The MZBR is a habitat to diverse and unique flora and fauna which contributes significantly towards the region's biodiversity. Terrestrial and aquatic flora and fauna species are found in the adjacent protected areas, i.e., Mana Pools National Park, Hurungwe, Chewore, and Charara safari areas. The MZBR valley floor area is endowed with diverse wildlife species including elephant (*Loxodonta africana*), buffalo (*Syncerus caffer*), black rhino (*Diceros bicornis*), painted wild dog (*Lycaon pictus*), nyala (*Tragelaphus angasii*), impala, kudu, waterbuck, zebra, hyena, and escarpment sable [35]. Nyamakate and Chundu Communal Areas are located within a predominantly Miombo woodland characterized by broad-leaved deciduous *Brachystegia*, *Julbernardia*, and *Isoberlinia* species.

### 2.2. Data Collection

The study used the mixed methods approach where a household questionnaire survey, key informant interviews, and focus group discussions were used to collect data on the causes, trends, and indicators of climate change. Prior to the survey, written annual permission to carry out the research was sought and granted from Hurungwe Rural District Council in 2015 and 2016. The research was approved by the Senate Research Council at the Chinhoyi University of Technology (CUT), Zimbabwe. In addition, all participants gave verbal informed consent to participate in the research. The study was ethically cleared by the CUT Ethics Committee.

Household surveys were used to collect quantitative data on climate trends. The household questionnaire contained questions related to (i) demographic profile of the household; (ii) perceptions towards climate change trends, impacts; and (iii) coping and adaptation strategies. The questions comprised both close-ended and open-ended questions. The household survey was carried out in the two communities between August 2015 and October 2016. The questionnaire was pre-tested with 16 households from Lima village located adjacent to Charara Safari Area to improve validity and reliability of the instruments. The questionnaires were revised after a pilot test to remove ambiguities and misunderstandings. Villages which are located close to the Hurungwe Safari Area were purposively sampled for the survey. Village registers which were obtained from the village heads were used to come up with the representative sample for the survey. Every third household was systematically sampled on the ground for the survey. Interviews were conducted with the head of the household or their spouse if they were not available. The geographical location of each sampled household was captured using a Geographical Position System Garmin Model GPS Map 64 (2013) and recorded.

Household questionnaires were administered to 320 people, of which 30% (*n*=96) were from five villages (India, Golf, Village 20, Murimbika, and Hotel) located in Nyamakate Resettlement Area, while the remaining 70% (*n*=224) were from Kabidza and Mayamba villages in Chundu Communal area. The proportion of respondents from each area ward is proportional to the number of households in the sampled villages. We selected only respondents that were more than 20 years since we assumed that they were more familiar with the local environment. The overall response rate was 100% since research assistants administered the questionnaires. Many of the interviewees (85%) were local farmers in the two areas. Socioeconomic and demographic profiles of the respondents are presented in Table 2.

Twelve (12) key informant interviews were also held with traditional leaders, ward councilor, and village elders. The structured key informant guide contained open-ended questions on traditional climate and weather indicators and prediction tools. During 2015 and 2016 survey, traditional leadership accompanied the researcher to their gardens, agricultural plots, and forest areas to identify biotic climate predictors and to learn about their use and their purpose in climate prediction and adaptation. This information together with data from the household survey and focus group discussions allowed the researcher to compile an inventory of flora and fauna species used in climate prediction.

To ensure triangulation of findings, focus group discussions (FGDs) were held to share, validate, and explore the findings of the household survey in greater detail [36]. A random call of at least two household survey respondents for participation in FGDs was made in each village. The FGDs were composed of between 8 to 15 farmers from mixed gender and/or separate male and female participants. During the FGDS, participants were asked to describe the causes of climate change and historical climatic events. Participants deliberated among themselves and reached a consensus before the final ranking was recorded. Challenges related to domination of the discussion by a few participants [6] were overcome by encouraging all group members to provide answers. Thus, the researcher elicited responses from all FGD participants in the listing and ranking of stressors [6].

### 2.3. Data Analysis

Data collected through the household questionnaire survey were coded by assigning numerical codes to text and then entered into Statistical Package for Social Sciences (SPSS software IBM Version 20, Chicago, USA) for analysis. Descriptive statistics (frequencies) were used to summarize demographic and socioeconomic data from the questionnaire response data set. Although the demographic profile (Table 1) shows distribution of respondents from the Resettlement and Communal Area, for further analysis, all the data were lumped and treated as a single data set. The percentage of all respondents in the household survey (*n*=320) that had perceived specific changes in the climate was calculated. One-way analysis of variance was used to test whether there is any significant difference in the mean respondents' perceptions towards awareness of changes in climate as well as temperature and rainfall changes. Trends in meteorological data, specifically rainfall and temperature, were analysed using Microsoft Excel 2007 [37].

Transcripts from key informant interviews and focus group discussions were translated to English and analysed through content analysis by identifying recurring themes, concepts, patterns, trends, and key words [38]. During qualitative content analysis, LEK on impacts of climate change was extracted and synthesized. According to Braun and Clarke [39], the deductive thematic analysis is a qualitative data analysis approach which is based on themes which are predetermined by the researcher's theoretical or analytic interest in the research area and is more explicitly driven. Data were therefore classified according to local observations of climate change, impacts of climate change on the biophysical and socioeconomic systems, and the coping and adaptation strategies.

## 3. Results

### 3.1. Local Community Perceptions on Climate Change and Variability

#### 3.1.1. Local Awareness of Climate Change and Vulnerability

Findings from key informant interviews indicated a general awareness among the village elders and other community leaderships that climate change and variability have been a reality in the area. One of the key informants who had stayed in Chundu Communal Area for over five decades (50 years) had this to say:

“I was born in this place in 1965 and my parents were also born here. Yes, I have heard of climate change. I can witness that the climate is changing judging from the shifting rainfall patterns, it is increasingly becoming erratic and local spirit mediums have advised that rainfall will decrease. We have observed it to be true and even the radio confirms this notion about declining and erratic rainfall.”

Perceptions of key informants are similar to about 58.1% (*n*=186) of household questionnaire respondents who indicated that they were aware of climate change whilst 41.9% (*n*=134) were not aware. Results from one-way ANOVA show that there were no significant differences in the mean responses across the entire sample (*p* ≤ 0.000). Focus group discussions further revealed that, in the study area, rainfall has been generally decreasing whilst temperature has been increasing. Key informants and FGD participants expressed concern that while the rainfall amount has been generally decreasing, the seasonal distribution of the rainfall was not even throughout the growing seasons.

#### 3.1.2. Patterns and Trends of Climate Change and Variability

**Figure 2:**
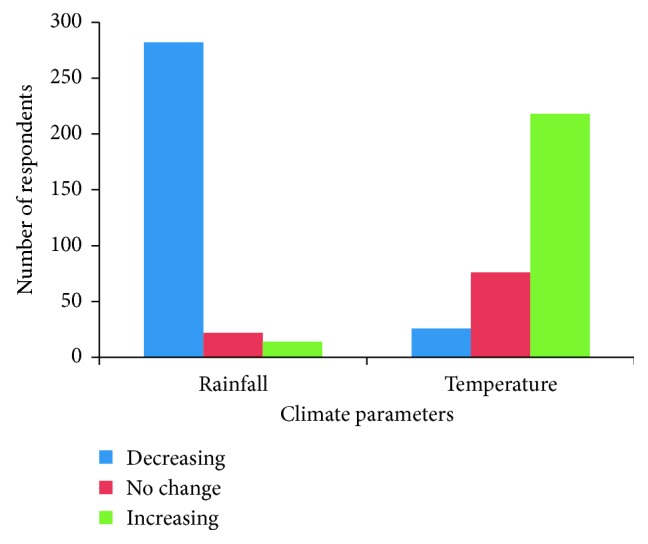
Household respondents' perceptions of rainfall and temperature (1980–2015).

**Figure 3:**
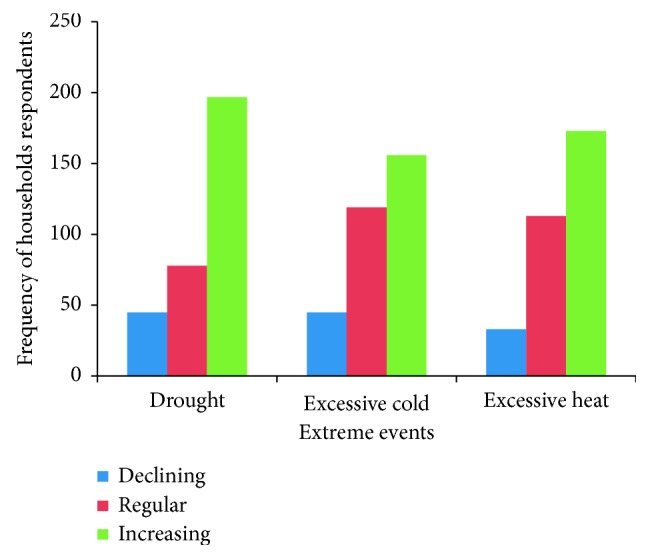
Household respondents' experiences of extreme events (1980–2015).

Results from the household survey show that the majority (88.1%; *n*=282) of the respondents perceived that the rainfall amount was generally decreasing whilst temperatures were increasing (68.1%; *n*=218) (Figure 2). Respondents showed mixed perceptions on temperature trends with 68.1% (*n*=218) of the respondents perceiving an increase, 8.1% (*n*=26) perceiving a decline, and 23.8% (*n*=76) perceiving that temperatures had remained the same (*P*=0.02) (Figure 3). The mean response was 1.40. On the other hand, no significant differences were observed on perceptions on rainfall with 4.4% (*n*=14) of the respondents perceiving an increase, 88.4% (*n*=282) perceiving a decline, and 6.9% (*n*=22) perceiving that temperatures had remained the same (*P*=0.03). The mean response was 2.83.

Findings from key informants and FGDs indicate that there has been a shift in the onset of rain season from October to mid-December whilst the end of rainy season has shifted from March to April since 2013. Traditional leadership noted that the community used to receive early rain like “*bumharutsva*” and “*gukurahundi*” prior to the onset of the rain season in November. The majority of key informants indicated that the onset of the rain season had shifted and was now shorter whilst the amount of rainfall has been declining. One key informant stated

“Rains are no longer coming in November but mid-December and end in early March. In the past, we used to get rainfall from October/November until around March/April. Overall, the length of the rain seasons has also decreased we only get rainfall for just two months or even one month. From 1982, we have been receiving normal rainfall except for 1992, 2001 and 2008 when we experienced severe drought. From 2008 up to now it has drastically decreased.”

A large proportion of the household respondents (94.4%; *n*=302) perceived that they had experienced drought as the most frequent extreme event followed by extreme heat (74.7%; *n*=239) (Figure 3). Key informants and FGD participants also confirmed that there have been changes in rainfall amount and temperature. One key informant stated

“2015/16 summer season has been the worst in terms of excessive heat. There were 2 days on a weekend that were the hottest ones we have ever seen. On those same days, our soya bean crops actually dried up within hours from the excessive heat. I think that temperatures at that time were over 40°C. On the other hand, we also experienced extremely cold periods in winter.”

A large proportion (94%; *n*=302) of the household respondents had experienced drought. Respondents showed mixed perceptions on the frequency of occurrence of drought with 61.6% (*n*=197) of the respondents perceiving an increase, 14% (*n*=45) perceiving a decline, and 24.4% (*n*=78) perceiving that temperatures had remained the same (*P*=0.01). On the other hand, no significant differences were observed on perceptions on excessive heat and excessive cold (*P*=0.07). Key informants and FGD participants mentioned that the area had experienced droughts during the following years: 1981/82; 91/92; 87/88; 2001/02; 2007/8; 2013/14. Approximately half of the household respondents (56%; *n*=178) stated that they had experienced extreme cold winters since 2008. Trends in the occurrence of cold winters were also perceived to be on the increase (48/8%; *n*=156), and the severity was moderate (43.4%; *n*=139). FGD participants also stated that, during the 2012/13 and 2014/2015 rain season, the area had received unusual hailstorms associated with destructive winds. However, findings from the household survey show that floods (3.8%; *n*=12) and tropical cyclones (10%; *n*=32) are not a common event in the area. All the key informants and FGD participants concurred that generally, weather conditions had become drier and rainfall timing was becoming more unpredictable.

Key informants were of the opinion that climate change is caused by industrial pollutants and the abandonment of traditional culture and practices. Traditional leaders noted that local chiefs generally no longer perform the traditional rainmaking ceremonies. It was reported that the chiefs could not perform the ceremonies because they do not qualify since these days people use various deviant acts to become chiefs. One key informant stated

“Back in the days when industries were few, we had no issues of climate change. The spirit mediums tell us that in terms of lifestyle, people used to be well behaved long ago and there were no cases of incest. Bereaved families did not store or hang up dead peoples' clothes (kuturika matata) like what people are doing nowadays. The ancestors are angered by these sins and in turn do not bless the area with rainfall.”

Traditional key informants also lamented that modern religions were overriding cultural practices most probably due to diverse cultural backgrounds of people located in Nyamakate Resettlement Area. Traditional leadership also highlighted that contemporary religious practices particularly Christianity of the apostolic sect seem to be in conflict with traditional values as indicated by invasion of sacred sites such as Hurungwe Mountain where they have established their shrines. Generally, all key informants stated that climate change is caused by excessive deforestation whilst other thought it is due to natural causes. Those who mentioned deforestation attributed this to clearance of land and wood harvesting for tobacco farming and curing, respectively.

### 3.2. Temperature and Rainfall Trends from Meteorological Data

**Figure 4:**
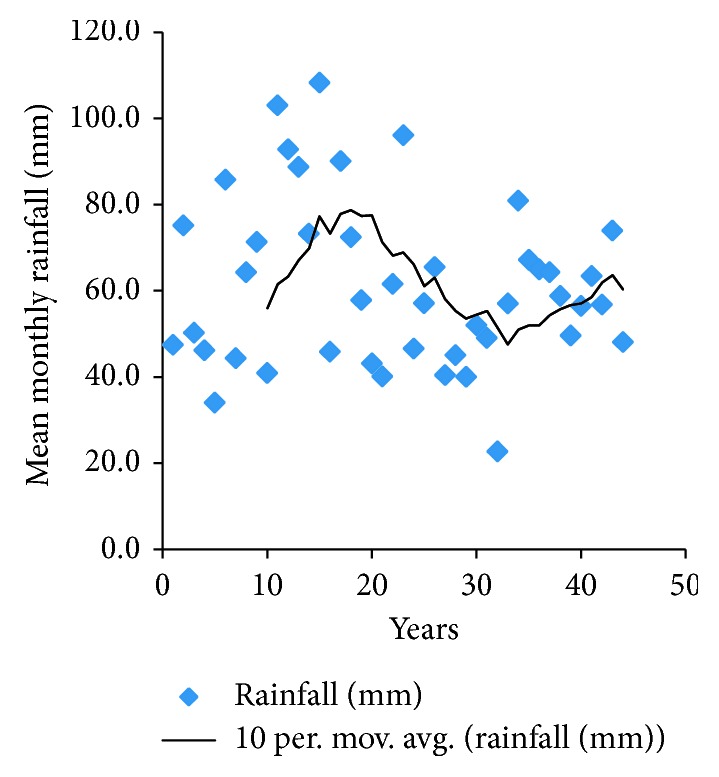
Mean monthly rainfall for Kariba (1967–2007) (source: Meteorological Services Department (MSD) Kariba Station).

Analysis of available rainfall data (Figure 4) from the Meteorological Services Department (Kariba Station) for the period 1976 to 2011 shows a slight decreasing trends in total rainfall for the period. The timing and transitions of seasons have been highly variable. Periods with very low rainfall include 1991/92 and 2001/02 seasons, corresponding to those years cited by all household survey respondents, key informants, and FGD participants.

**Figure 5:**
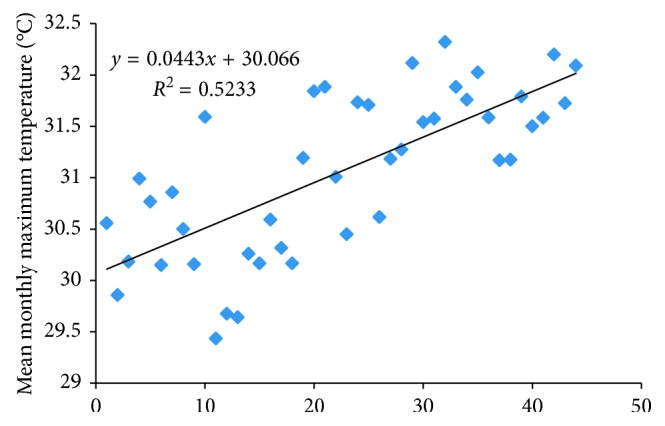
Mean monthly temperature for Kariba (1967–2007) (source: Meteorological Services Department (MSD) Kariba Station).

Mean monthly minimum and maximum temperatures for Kariba Station (1976–2007) show a general increase (Figure 5). The period 1970 to 1981 recorded 30.2°C as the average maximum temperature.

The ten-year period, 1981–1990, shows a significant temperature increase with a calculated mean average maximum temperature for this period as 31.4°C, showing a 1.2°C increase from 30.2°C. Ever since 1991 to date, temperatures have been increasing, particularly a record of 33.1°C for the year 2007. Thus, in Kariba, temperatures are increasing.

### 3.3. Impacts of Climate Change and Variability on Livelihood Systems

Household respondents, key informants, and focus group discussants were aware of the impacts of climate change on socioeconomic and biophysical components of the environment (Table 3). The changing climate has resulted in a general decline in agricultural productivity, including changes in the availability of ecosystem goods and services. About 43% (*n*=139) of the respondents indicate that they had experienced livestock diseases such as red water due to climate change. Approximately 59% (*n*=189) attributed the decrease of pastures to drought which ranked third after increase in livestock and population increase. About 8% (26) households had lost at least two cattle (currently valued at approximately US$ 800) due to drought.

A large proportion of household respondents (78%; *n*=250) cited declining rainfall (53%; *n*=168) as the major factor contributing to water shortages whilst another proportion (43%; *n*=136) also indicated that disappearance of wetlands (68; *n*=218) was also mentioned to be succumbing to decreasing rainfall. Field observations revealed that some farmers cultivated in wetlands and valley bottoms, taking advantage of residual moisture in the soils. Personal field observations revealed boreholes, rivers, streams, and wells as the key water sources in the area. Key informants indicated that there have been substantial changes to the flow regime of streams such as Chitake, Chewore, Kabidza, Chitake, Mvurameshi, Mvuramachena, Samhofu, and Hodobe. For instance, key informants reported that Rukomechi river flow regime had changed from perennial to seasonal since 2015/2016 season. The study villages used to have several water sources, which would supply water throughout the year such as natural springs and wetlands, but most of them have dried up.

Key informants and FGD participants identify wild fruits and forest products, which they used to sustain livelihoods during periods of drought. Respondents indicated that *Piliostigma thonningii* (Schumach) fruits were being harvested and pounded into powder to cook porridge, whilst *Diospyros mespiliformis* (mushuma) and *Parinari Curatellifolia* (muchakata) fruits are used as an alternative food source during drought. In addition, exotic tree species such as raw mangoes have also been harvested prematurely and cooked for consumption during the 1991/92 and 2007/8 drought period. During drought local communities also rely on indigenous shrubs and underground tubers such as air potato (manyanya) and “mupama”, although even these roots only do well when there is good rainfall. *Dioscorea praehensilis* Benth (mupama) and the air potato *Dioscorea bulbifera* (*manyanya*) are woody perennial plants, which both belong to the family Dioscoreaceae. The plants produce edible underground organs (tubers). The air potato (mupama) is one of the most widely consumed yam species. Mupama tree is also used during drought periods. For example, respondents mentioned the tree species provided food relief to most vulnerable and poor households during the most severe 1991/2 and 2007/8 drought period. Respondents stated that the yam plant produces potato like roots are edible. Unlike the ordinary potato and sweet potato tubers, the air potato tubers have to be thoroughly boiled to remove the bitter taste before consumption. Other key informants reported that if the tubers are underprepared, their consumption could lead to severe stomach ailments and eventually death.

Key informants reported that *Rhynchosia venulosa* (mukoyo) is as one of the popular drought relief plant species among the local community members. Key informants and FGD participants stated that traditional beer-brewing experts use the roots of the legume to brew beer, which can be sold locally or taken to the border town of Chirundu for sale. Key informants suggested that commercialization of the by-product from the legume is important in contributing towards household income during drought periods. The shrub has been cited as one of the underutilised legumes which have tremendous potential for commercial exploitation in the area. Participants reported that they even illegally harvest the plant in the adjacent protected area since the species is already disappearing in nearby community forests.

A few respondents cited bee farming as one of the key coping strategies in response to changing climatic shortages. The beehives are made from mupfuti tree timber. Upon completion, the beehives are placed in the mupondo or mutsabvi tree because it has flowers, which easily attract the bees towards the hives. Local community members practice highlighted that bee farming requires patience and dedication; hence, very few people use it as a coping strategy. The next section presents an analysis of factors which influence the choice of coping strategies in the study area.

## 4. Discussion

### 4.1. Local Community Perceptions on Trends and Causes of Climate Change

Findings from this study indicate that local communities perceive that rainfall is decreasing whilst temperatures are increasing. Findings are in line with IPCC [40] predictions that temperatures across different scales are set to increase whilst rainfall in southern Africa is set to decrease. Results from this study show that scientific rainfall data which show interannual variations and a general decline in rainfall for over the past 30 years corroborate with the people's perception of climate changes. Farmers' perceptions of climate change in the study area are in line with empirical data and other studies on farmers perceptions inhabiting marginal areas in Zimbabwe [23, 41, 42]. Findings are also similar to other studies in Zimbabwe such as Guruve District where Gwenzi et al. [43] and Hwedza and Makoni [44] have reported unpredictable rainfall, declining rainfall, and increasing temperatures as some of the indicators of climate change. Local ecological knowledge on climate patterns and impacts has been documented in other studies in different parts of Africa [45–48]. Using IKS, Nkomwa et al. [22] reported that farmers in Malawi observed delayed and unpredictable onset of rainfall, declining rainfall trends, warming temperatures, and increased frequency of dry spells as some of the key indicators of a changing climate. However, there are variations with respect to observed extreme events. In this study, respondents cited frequent droughts and heat waves as the common extreme events. These findings are similar to IPCC [40] report, which indicated that persistent droughts and extreme temperatures are some of the indicators of a changing climate. Whilst other studies have observed extreme events such as cyclones and floods [49–51], in this study, farmers did not identify such events. In Zimbabwe, two cyclones Elline (experienced in 2000) and Japhet (experienced in 2003) have mainly affected the Manicaland and Masvingo provinces [51], which the study respondents have not experienced. According to the [52] the major flood-prone areas in Zimbabwe are selected parts of the southern lowveld and the lower Zambezi valley, that is Muzarabani, Middle Sabi, Tsholotsho, Malipati, Chikwalakwala, and Tuli-Shashe.

In this study, farmers attribute climate change to both anthropogenic and spiritual causes. This is in line with findings by [53] who noticed that farmers in Ghana perceived that disasters such as prolonged droughts are inflicted by spiritual factors such as the gods. Our findings also correspond with the IPCC [40] report that combinations of anthropogenic and natural forces are the major cases of climatic changes.

### 4.2. Local Ecological Knowledge on the Impacts of Climate Change

This study found that community members agreed that extreme events related to climate change such as prolonged dry periods and excessive temperatures have affected agricultural activities and the biophysical environment. Nyikahadzoi et al. [25] note that climate change is expected to continue to pose a serious threat to agriculture in southern Africa as annual rainfall amounts are expected to decline and temperatures are expected to increase. Climate change has led to highly variable yields in arable agriculture (both rain-fed and irrigated) in African countries [54]. Findings in this study are in line with the IPCC [40] which states that climate change impacts affect both natural and human systems. Respondents indicated that climate change has affected seasonal rainfall patterns by reducing the length of the rainy periods as well as the amount of rain with consequences on crop and livestock production. Similar observations by Chikozho [51] are that low and erratic rainfall is leading to low and unpredictable levels of crop production in the semidry agroecological zones of Zimbabwe. Study findings revealed that some farmers resorted to stored grain for seed but did not indicate any loss of seed variety impacts associated with climate change. This contrasts with findings by Mburu Gathuru and Kaguna [55] who revealed that, in Kenya, climate change, mainly reduced rainfall levels, has led to the disappearance of some of the indigenous seed varieties as continued failure of crops is affecting the capacity for production of good seeds. In this study, farmers indicated that the planting season has been shortened due to shifting in the timing of the onset of the rain season. Findings in this study are in line with Mburu Gathuru and Kaguna [55] who observed that, in Kenya, climate change has caused disruption of the planting calendar such that it has become increasingly difficult for youthful farmers to predict seasonal regimes.

Findings on the negative impacts of climate change on pastures and livestock health concur with those by Hopping et al. [56] who reported that pastoralists in the Tibetan rangelands, China, have the same opinion that changing climate is driving undesirable trends in grassland and livestock health. Rose et al. [57] also noted cases of decline in pastures due to low and erratic rainfall. Other authors have also noted that livestock production faces the problem of poor and variable rangeland productivity and desertification processes [24, 58, 59]. Generally, reduction in fresh water resources affects natural resource and climate-dependent sectors such as forestry, agriculture, water, and fisheries [60]. Findings from this study indicate that local communities use LEK to detect changes in water resources due to climate change and variability. Respondents indicated that frequent extreme events such as drought and increasing temperatures affect soil moisture and surface water availability for both domestic use and agriculture. This is in line with United Nations Framework on Climate Change [61] assertion that Africa faces challenges related to water availability and spatial variations in the location and need for water resources. Observations from this study are also similar to those by Taylor et al. [62] who noticed that more frequent and intense climate extremes such as droughts and floods increase variability in soil moisture and surface water. Observations in this study related to drying up of rivers and poor water quality in surface and groundwater systems coincide with findings by Urama and Ozor [63] who reported that impacts on water resources act in conjunction with other factors to affect ecosystem health and socioeconomic well-being of human communities. For example, Mburu Gathuru and Kaguna [55] also noticed that, in Kenya, climate change interacts with anthropogenic activities along rivers to contribute to reduction of river water volume over time and weakening of critical ecosystems like forest watersheds.

Farmers at the agriculture wildlife interface notice impacts of climate change on wildlife resource abundance such as disappearance of wetlands, habitat changes, and changes in the phenology of indigenous tree species. These observations are in line with Dube and Phiri [64] who reported that climate change is affecting biodiversity in Zimbabwe as indicated by the disappearance of natural habitats, flora, and fauna. In Tanzania, Paavola [65] also noticed that forests, wildlife, and wetlands are being impacted by climate change. Farmers also reported that although wildlife resources such as fruits are consumed to avert food shortages during drought, their abundance has declined due to climatic changes. Results from this study agree with those by Rurinda et al. [44], who reported that the availability of wild fruits and social safety nets was affected directly and indirectly by extreme temperatures and increased rainfall variability, impacting on the livelihoods of resource-constrained farmers. Similar observations were also seen in [66] who noticed that farmers in Makonde district, Zimbabwe, perceived changes in ecosystem productivity, goods, and services as a result of climate-induced factors. Nhemachena et al. [41] also indicated that the phenology of indigenous fruit trees and invertebrate species was under threat from climate-induced changes on water availability and wetlands. However, in this study, respondents did not identify any impacts associated with invertebrate species.

The recruitment of plants has been constrained by low rainfall and temperature leading to low productivity [67]. Low productivity of primary producers (plants) had severe cascading effects on both domestic and wildlife species along the food chain. Climate change has led to creation of strong tensions between humans and wildlife species due to shortage of resources (especially food and water). Occurrence of habitat patches and invasive species (which are inedible to animals) led to animals exceeding their home-ranges and encroaching human habitats, thereby causing damage to crops and property paving a way to human-wildlife conflict. Climate change has the potential to alter migratory routes (and timings) of species that use both seasonal wetlands (migratory birds) and track seasonal changes in vegetation (herbivores) [68]. This increase conflicts between people and large mammals such as elephants, particularly in areas where rainfall is low. Change [69] states that wildlife-farming conflict was potentially exacerbated by climate change, in particular, drought, which encourages wildlife to forage on farmlands.

In this study, respondents also noted that climatic parameters interact with other nonclimatic factors such as illegal harvesting and pressure from human population increase also influence the abundance of wildlife resources. For instance, trees belonging to the dominant family within the Miombo region are threatened by other nonclimatic factors such as deforestation. Similar observations have been made in Mozambique where trees associated with Miombo woodland utilised for traditional purposes are declining due to overexploitation and destructive collection [70]. Mubaya et al. [71] suggest that although climatic factors are critical in determining production in agroecosystems, multiple stressors interact to influence the abundance and diversity of natural resources.

### 4.3. Coping and Adaptation Strategies

Findings from this study revealed that local communities use LEK on wildlife resources, water conservation, indigenous plant food sources, and alternative income generation as way of adapting and coping with changing rainfall patterns, extreme temperatures, and droughts. Local communities use water harvesting such as digging wells, which collect water within wetlands during the rainy season, as one of the key strategies of coping with water shortages. The water is then used for supplementary irrigation of vegetables and crops during the dry season. Similar observations have been made by Van Campenhout et al. [72] that, in the dry season, farmers use the water conserved in the wells and basins for irrigation. Home gardens are widely done in southern Africa especially Zimbabwe, Mozambique, Botswana, and South Africa where there is growing of food crops like maize, rice, vegetable, and fruits for barter trading and income generation [72]. For example, in South Africa, indigenous people grow cash crops like vegetables, tomatoes, and maize in home gardens to increase household income [73].

This study established that illegal harvesting of wildlife and harvesting and consumption of wild fruits and legumes can alleviate food shortages during drought. Similar findings have been observed by [74, 75] who noted that off-farm income derived from exploitation of wildlife resources is critical to livelihoods and overall adaptive capacity. In coping with risk due to excessive or low rainfall, drought, and crop failure, some traditional people in Ghana also supplement their food by hunting, fishing, and gathering wild food plants [76]. Findings from this study on the use of edible tubers from family Dioscoreaceae are similar to findings elsewhere in Africa. Similar sentiments have been expressed by Mortimore and Manvell [77] who reported that small holder farmers in northern Nigeria making use of biodiversity in cultivated crops and wild plants as one of the adaptation strategies. For example, Bruschi et al. [70] found that some plant species like *Dioscorea cochleari apiculata* and *Dioscorea dumetorum* have been collected from a Miombo woodland and eaten as a means of averting food shortages during drought periods in a rural community of Muda-Serraçã, central Mozambique. Similarly, in this study, the same species provide alternative food during drought periods. In addition, both studies acknowledge that utilisation of the tubers requires one to be thoroughly acquainted with the skills and techniques for making some of the poisonous wild plants edible. Apart from southern Africa, the edible legumes are widely known as famine foods in East Africa and have also been reported as cultivated in some parts of West Africa [78]. Key informants also emphasized that *Dioscorea cochleari apiculata* and *D. dumetorum* may be eaten only after they have undergone appropriate preparation. Basically, the preparation procedure for the tubers involve the following: peeling the tuber, cutting it into thin slices, dry and wash several hours in a river, always changing the place, and then boil thoroughly for a prolonged period of time till they are cooked. Failure to do this may cause vomiting and even death as revealed by observations from East Africa [79].

In this study, few individuals indicated that they resort to production of handcrafts for sale as a strategy to increase household income. Mogotsi et al. [80] concurs that local communities have engaged in different living strategies like producing crafts for selling for income generation in order to reduce poverty and starvation during drought periods. For example, in Zimbabwe and South Africa, they use murara (*Hyphanate petersiana*) leaves for basket weaving and production of wine from the sap [24, 81]. In Zimbabwe, njemani production has become a source of living for many people in Sengwe area and some of people no longer get involved in farming [82]. Similarly, in this study, some farmers indicated that they used local plant resources to weave mats, curve wood crafts, and brew beer for selling to boost household income but still engage in farming. However, unlike other communities who use grass plants like *Hyphanate petersiana* to brew local beer [82], in this study, they used legume *Rhynchosia venulosa* to brew beer.

## 5. Conclusion

Local communities within the MZBR perceive climatic changes especially changing rainfall and temperature. In addition, they perceive cultural and anthropogenic factors such as deforestation as some of the causes of climatic change. The community uses ethnobotanical and ethnozoological knowledge to detect weather changes. Local communities in biosphere reserves have noticed impacts of climate change on the socioeconomic and biophysical livelihood assets. Consequently, they have developed several livelihood coping and adaptation strategies to enhance the food security of their families in response to the changing climatic conditions. Land tenure category influences the choice of drought-related coping strategies for local communities in the MZBR.

Based on the findings, local ecological knowledge can provide information on the changing climate especially in under-researched areas such as the MZBR. Such information can complement scientific data to inform policy on best practices to build adaptive capacity of rural communities within biosphere reserves in semiarid tropical savanna. LEK, in particular, traditional phenological knowledge (TPK), can be adopted to complement scientific forecasts especially under situations where the local community recognizes that climate is changing. Findings from this study indicate that local ecological knowledge can provide information on household livelihood strategies under a changing climate especially in under-researched areas such as the MZBR. Such information can compliment scientific data to inform policy on best practices to build adaptive capacity of rural communities within biosphere reserves in semi-arid tropical savanna. Integrating local ecological knowledge into climate change adaptation and biodiversity conservation is possible if the knowledge holders are directly engaged as active participants in these efforts. Results from this study highlight the need for harnessing local knowledge to enhance community resilience and promote ecosystem-based adaptation strategies in the face of a changing climate.

## Figures and Tables

**Table 1:** Attributes of the study sites within the transition zone of the MZBR.

Attribute	Nyamakate Resettlement Area	Chundu Communal Area
Land use status	Planned Resettlement Area	Traditional Communal Area
Ownership	Government	Government
Management	Public	Public
Key stakeholders	Hurungwe Rural District Council	Hurungwe Rural District Council
Conservation initiatives		
Year established	1980	1958
Size (ha)	110145	60753
Fauna	Domesticated animals	Domesticated animals
Flora	Dry savannah dominated by Miombo woodland	Dry savanna dominated by Miombo woodland
Climate	Marked seasonal annual rainfall 700–800 mm high means monthly temperatures approximately 40°C and average minimum temperatures are around 10°C	
Human population	High (over 13000 small-scale subsistence farmers located in designated villages)	High (communal farmers in a typical rural setup villages)
Forms of tourism	Nonconsumptive tourism	Nonconsumptive tourism
Source of livelihoods for local communities	Semisubsistence agriculture, Communal Area Management Programme for Indigenous Resources (CAMPFIRE)	Subsistence agriculture, CAMPFIRE
Human-wildlife conflicts	Human carnivore conflict	Human carnivore conflict

Source: Chimhowu and Hulme [32]; Mbereko et al. [33]; Madhekeni and Zhou [34].

**Table 2:** Socioeconomic and demographic profiles of respondents.

Household characteristics		Study sites		Total	%
		Nyamakate (*n*)	Chundu (*n*)		
Gender of household head	Male	64	171	235	73
	Female	32	53	85	27

Age of the household head	Below 29	24	20	44	14
	30–40	34	75	109	34
	41–50	14	32	46	14
	51–60	13	26	39	12
	60+	11	71	82	26

Marital status of the head	Never married	5	7	12	4
	Married	73	180	253	79
	Divorced/separated	5	7	12	4
	Widowed	13	28	41	13
	Cohabiting	0	2	2	1

Household size	1–3	11	32	43	13
	4–6	49	113	162	51
	7–9	25	58	83	26
	10–12	9	17	26	8
	Above 13	2	4	6	2

Period of stay in the MZBR	1–5	20	18	38	12
	5–10	16	24	40	13
	10–15	15	26	41	13
	16–20	26	33	59	18
	21–25	6	22	28	9
	26–30	6	35	41	13
	Above 30	7	66	73	23

Education level of household head	None	11	54	65	20
	Primary	28	85	113	35
	Secondary	55	82	137	43
	Tertiary	1	1	2	1
	Vocational training	1	2	3	1

Wealth rank category	Poor	20	80	100	31
	Average	68	127	195	61
	Rich	8	17	25	8

Occupation/job of the household head	Gardening	17	11	28	9
	Rural farmer	73	199	272	85
	Farm laborer	1	3	4	1
	Business	2	3	5	2
	None	2	0	2	1
	Pension	0	2	2	1
	Professional	0	6	6	2

Family member employed in the wildlife sector	Yes	12	15	27	8
	No	84	209	293	92

**Table 3:** Local community perceptions of climate change impacts on livelihood systems.

Perceived climate trend	Impacts on livelihood system	Coping and adaptation strategy
Agricultural activities		
Declining and erratic rainfall shifting rain season	Inadequate moisture for plants production (BI) Decrease in crop productivity, e.g., maize (SE) Changes in crops/varieties Short and unpredictable planting season (SE) Increased prevalence of new pests and diseases (BI/SE)	Cultivate in wetlands and low-lying areas Change crop variety from long season to short season (A) Use stored grain as seed reduced the overall area under cultivation
Extreme temperatures (heat waves and very cold winters)	Wilting of maize and tobacco has mostly been affected by excessive heat (BI/SE)	Water conservation techniques such as conservation agriculture and mulching
Persistent droughts	Household food shortages due to poor harvest/low agricultural output (SE)	Harvest wild fruits, e.g., muchekecha, and wild legumes such as *Dioscorea praehensilis* Benth (mupama), and the air potato, *Dioscorea bulbifera* (manyanya), and *Rhynchosia venulosa* (mukoyo) during drought periods Harvest wild animals, e.g., rabbits, warthogs, and mice, and community/nutritional gardens Off-farm jobs in nearby commercial farms Cooking raw bananas and mangoes Gold panning, Selling livestock. Barter trading, for example, exchanging 2 gallons maize with a goat
	Reduced household income (SE)	Rearing of domestic guinea fowls (*Numida meleagris f. domestica*) and rock hyrax (*Procavia capensis*) for sale; bee-keeping informal trading; food for work; weaving and hand crafting; Off-farm activities, e.g., seek employment in nearby farms, and migrant labor in Zambia
Reduced rainfall and excessive heat drought	Deterioration on quantity and quality of livestock grazing areas (BI/SE) Reduced livestock, e.g., cattle and goats, and reproductive rate and capacity has been affected (SE) Increase in roadrunner mortalities and reduced reproduction (SE) Increase in cases of climate-induced disease outbreaks (SE) Shortage of water for livestock (BI); livestock mortality (cattle, goats, and sheep); livestock diseases (e.g., red water in sheep, goats, and cattle), and deteriorating health condition (all domestic animals) (SE/BI)	Livestock graze within wetlands and adjacent protected area Store maize crop residue for cattle feed Reduce livestock numbers

Water resources		
Reduced rainfall and high temperatures	Reduction in water sources due to drying up of water sources boreholes; domestic wells drying up before the end of the next rainy season (BI/SE) Change in river flow from perennial to seasonal (BI); disease outbreaks such as headache, malaria, and diarrhoea (SE) Lack of water for setting up tobacco seed beds (BI/SE) Wetlands drying up	Dig deep wells along river beds and on wetlands Women travel long distances to fetch water Several households (e.g., up to 44) share same borehole

Forest resources		
Reduce rainfall	Changes in tree phenology (both domestic and exotic tree species), e.g., mazhanje and mango (BI) Prolonged leaf senescence time in leaf fall for deciduous trees from August to October, tree leaves would be green but now the leaves have actually fallen off when they used to fall off only in August, e.g., mupfuti, munondo, mutsonzowa, mukonono, mutowa (BI/SE) Most indigenous and exotic fruit trees including fruit trees are no longer producing fruit disappearance of reduced fruit production, e.g., *Diospyros mespiliformis* and muhacha (BE/SE)	Planting of indigenous and exotic tree species
Extreme temperatures (heat waves and excessive cold)	Premature drying up of fruits like nhunguru (BI)	Planting indigenous and exotic trees

Soil resources		
Reduced rainfall and excessive temperatures	Soil carbon stocks have been disturbed (BI) Change in soil quality over time (BI)	Conserve our soils through the use of “madhunduru”; apply fertilizers

Wildlife resources		
Drought (2002–2008)	Habitat encroachment, e.g., human expansion of cultivation into buffer zone (BI) Livestock depredation during the prolonged dry season; lions mostly follow after donkeys and cows; hyenas target goats wild animals; lions and hyenas attack livestock (BI) Crop destruction (buffaloes and elands destroy tobacco and eat maize in the fields during March, April, and May Bush pigs and baboons create a menace during the planting and harvesting season from December to May (SE)	Illegal hunting and harvesting

Key: Socioeconomic impact (SE); biophysical Impact (BI).

## Data Availability

Data can be made available subject to terms and conditions related to open-access publication.
